# Characterization and Study of Transgenic Cultivars by Capillary and Microchip Electrophoresis

**DOI:** 10.3390/ijms151223851

**Published:** 2014-12-22

**Authors:** Elena Domínguez Vega, Maria Luisa Marina

**Affiliations:** 1 Division of BioAnalytical Chemistry, AIMMS Research Group of BioMolecular Analysis, VU University Amsterdam, De Boelelaan 1083, 1081 HV Amsterdam, The Netherlands; 2 Department of Analytical Chemistry, Physical Chemistry and Chemical Engineering, Faculty of Biology, Environmental Sciences and Chemistry, University of Alcalá, Ctra. Madrid-Barcelona Km. 33.600, 28871 Alcalá de Henares, Madrid, Spain; E-Mail: mluisa.marina@uah.es

**Keywords:** capillary electrophoresis, microchip electrophoresis, transgenic cultivars, GMO, safety assessment, food

## Abstract

Advances in biotechnology have increased the demand for suitable analytical techniques for the analysis of genetically modified organisms. Study of the substantial equivalence, discrimination between transgenic and non-transgenic cultivars, study of the unintended effects caused by a genetic modification or their response to diverse situations or stress conditions (e.g., environmental, climatic, infections) are some of the concerns that need to be addressed. Capillary electrophoresis (CE) is emerging as an alternative to conventional techniques for the study and characterization of genetically modified organisms. This article reviews the most recent applications of CE for the analysis and characterization of transgenic cultivars in the last five years. Different strategies have been described depending on the level analyzed (DNA, proteins or metabolites). Capillary gel electrophoresis (CGE) has shown to be particularly useful for the analysis of DNA fragments amplified by PCR. Metabolites and proteins have been mainly separated using capillary zone electrophoresis (CZE) using UV and MS detection. Electrophoretic chips have also proven their ability in the analysis of transgenic cultivars and a section describing the new applications is also included.

## 1. Introduction

Advances in biotechnology have enabled the production of genetically modified organisms (GMOs) by the introduction of new DNA sequences to confer new functions or modify certain properties of the organism. In the particular case of the food industry, GMOs have played an important role and a number of transgenic cultivars have been developed in recent years. Initially, transgenic cultivars were developed with agronomic purposes, such as, for instance, to confer tolerance to herbicides (e.g., glyphosate, gluphosinate and oxinyl) or resistance to certain insects (e.g., *Bacillus thuringiensis*) [1]. Soybean, maize, canola and cotton are the main GM cultivars presenting tolerance to pesticides and resistance to insects. Nowadays, new advances in the production of GMOs are expected to bring some additional valuable properties to the cultivar and/or benefits for the consumer (e.g., rice enriched with Vitamin A and altered fatty acid composition) [1]. In addition, first GMOs were developed with single attributes while new GMOs mostly comprise combinations of two or more modifications. A clear example is the genetically modified corn SmartStax™. This GMO was introduced in the market in 2009 and contains a combination of six modifications that confer to the organism multiple modes of insect protection and herbicide tolerance [2]. As a consequence of the increasing value of the transgenic cultivars, a high number of foods produced from GMOs are currently available on the market. However, there is still controversy regarding their introduction to agriculture and their use in diet. Therefore, increasing research efforts have been focused on the evaluation of the risk of the use of GM cultivars.

Safety assessment is not an easy task especially in the case of GM cultivars due to their complex composition. The assessment of substantial equivalence has been introduced as a starting point for safety evaluation in genetically modified foods [3,4]. Substantial equivalence studies rely on the comparison of certain properties of a genetically modified food with the appropriate “safe” counterpart. Therefore, if no differences are found between both cultivars, it is assumed that the new food does not comprise any additional risk to health. Another aspect of serious concern is the possibility of unintended effects caused by the transgenic cultivars. Unintended changes can have different origins and can be produced at different stages of the cycle of the GMO [5]. For instance, GMO cultivars can have different responses under stress or certain situations (e.g., environmental, climatic, infections) than conventional ones [6,7].

Based on that, some countries have established regulations for labeling products containing transgenic cultivars [8]. European food legislation is particularly strict and each product containing more than 0.9% of GMO should be labeled as “contains GMO” [9]. Hence, reliable, low cost, accurate and fast analytical methods for the identification, characterization and study of transgenic cultivars and food-derived products are necessary. Different methods have been reported to meet these regulations and pubic requirements [1,10,11]. Overall, these methods are based on the detection of transgenic DNA [12,13,14] or particular proteins expressed by GMOs [15]. The polymerase chain reaction (PCR) and/or immunoassays such as enzyme-linked immunosorbent assay (ELISA) are the most widely employed methods for these purposes. Protein and/or metabolite profiling has played a fundamental role in the assessment of substantial equivalence and the study of unintended effects caused by a genetic modification [5]. Moreover, profiling techniques have proven their capability in the study of GMOs response to diverse situation or stress conditions (e.g., environmental, climatic, infections). Recently, mass spectrometry (MS)-based methodologies have also shown a huge potential for the study and characterization of transgenic cultivars [16].

Capillary electrophoresis (CE) represents an interesting alternative for the identification, characterization and study of GMOs. Its high speed, low sample and solvent consumption and low cost make this technique attractive for routine determination of GMOs in foods. Especially its high resolution power compared with traditional gel electrophoresis make CE ideal for the detection of DNA fragments obtained after amplification of the GMOs target sequences. On the other hand, CE has proven its potential for profiling providing complementary information with respect to other applied methodologies as liquid chromatography (LC)-MS [17]. Current trends in the use of high-performance separation methods for the analysis of genetically modified foods have been reviewed [18]. In 2009, an overview focused on the applications of capillary electrophoresis and microchip capillary electrophoresis to the detection of GMOs was published [19].

This review covers all the literature concerning the most recent applications of CE to the analysis and characterization of transgenic cultivars (from January 2009 to September 2014). The works included in this review have been classified based on the target analyte/s and the study of GMOs is based on their determination. According to this criterion, the review has been divided in three main sections: (i) DNA; (ii) protein; and (iii) metabolite determinations. Finally, the new applications of electrophoretic chips for the analysis of transgenic cultivars are also discussed.

## 2. Applications of Capillary Electrophoresis (CE) to the Study and Characterization of Transgenic Cultivars

### 2.1. DNA Analysis

DNA approaches are generally used for the determination of GMOs because of their increased specificity, sensitivity and robustness. With the exception of real-time PCR, amplified DNA fragments should be separated and detected after amplification. Agarose gel electrophoresis is the preferred technique for this purpose [20]. However, some of the drawbacks of conventional gel electrophoresis are the long analysis time, the tediousness of the procedure and the difficulty to identify heteroxygous. CE, in particular capillary gel electrophoresis (CGE), has proven to be an effective technique for DNA analysis as it provides higher separation efficiencies and sensitivity and offers short analysis time and small sample/reagent consumption (see Table 1). Especially in the case of multiplex PCR approaches generally employed for the assessment of GMOs, these characteristics are fundamental to properly separate and detect the multiple amplified DNA fragments. As fragments can be easily labeled with fluorescent dyes, CGE in general is combined with fluorescence detection in DNA analysis offering good sensitivity with limits of detection (LODs) ranging from 0.01% to 1% of GMO. For instance, Holck *et al.* reported a simple multiplex PCR-CE method for the qualitative screening of five maize events, namely DAS59122, LY038, MON88017, MIR604 and event 3272 and the maize endogenous *hmga* gene [21]. The *hmga* gene was employed as internal reference to correct differences in total maize DNA in samples. The approach was based on a hexaplex PCR method with fluorescent labeled forward primers (carboxyfluorescein (FAM), NED™ and VIC^®^) and unlabelled reverse primers, yielding labeled amplicons of specific lengths that are detectable by CGE with laser induced fluorescence (LIF). One aspect that must be taken into account in multiplex PCR is the possibility that differences in amplification efficiencies can result in different amplification rates of the targets. Therefore, a common nine nucleotide sequence in the 5'-end was included in all the primers in order to obtain similar amplification efficiencies as a result of the higher similarity of the primer sequences. The CGE separation and detection of the amplified fragments was performed using the performance optimized polymer-7™ (POP-7™) and separation was reached in 1800 s. The method was very sensitive allowing detecting up to 0.1% of each event for 100 ng of each GMO (~40 DNA copies) even in the presence of high concentrations of other templates. The developed method showed to be a simple, fast, high throughput and sensitive qualitative method for the screening of samples containing five different maize GMOs. In order to quantitatively determine these GMOs, the same group reported an alternative methodology based on competitive PCR and CGE-LIF [22]. Quantitative multiplex PCR methods usually are complex and offer low sensitivity. In this method, a simple and novel competitive multiplex approach was employed for the quantification of multiple DNA targets. The multiplex quantitative competitive PCR method was adapted from the previous qualitative PCR method for the same five maize events (DAS59122, LY038, MON88017, MIR604 and event 3272) and *hmga* gene [21]. In this case, the quantitative multiplex reaction was performed by adding competitors in equal known amounts as a restriction enzyme-digested plasmid insert. As the fragments have these same primer annealing sequences and similar sequence, each GM event and competitor is amplified with similar efficiency. Therefore, after CGE-LIF analysis, the relative amounts of GMO and GMO competitor (after correction for differences in maize DNA with *hmga* gene were employed to determine the amount of GMO. Limits of detection of 0.1% of each GM event (~40 DNA copies for 100 ng of template) were obtained with this methodology. In other work, the same group developed a quantitative multiplex ligation-dependent probe amplification (MLPA) method for the determination of eight GM events [23]. Ligation-based approaches combine a ligation step and an amplification step. In ligation-dependent probe amplification, the products resulting from the ligation of bipartite probes are amplified using universal amplification primers. Therefore, the same amplification efficiency is obtained for all the fragments. Other advantages of MLPA are the reduced interaction between probes, higher specificity and reproducibility. In addition, higher levels of multiplexing are possible with this approach [24]. In this work, amplified fragments for TC1507, MON810, NK603, MON863, BT176, T25, GA21, BT11 and the endogenous *hmga* maize reference gen were efficiently separated and detected by CGE-LIF. The CGE-LIF separation was performed using the POP-7™ polymer as capillary coating allowing good resolution of the nine target sequences that were detected with good sensitivity (0.1%–0.5% GMO). Quantification in the range of 0%–2% GMO was obtained by comparing the target GM fragment with the *hmga* internal reference gene signal. GMO quantification of samples with known and unknown GMO content was performed with the proposed method. Compared with quantitative real-time PCR, the same GMO content was determined for 149 of the 160 samples analyzed by MLPA-CGE-LIF. Multiplex ligation-dependent genome amplification (MLGA) and CGE with LIF detection have been also used for the determination of maize GMOs [25]. Contrary to MLPA, in MLGA the ligation of genomic DNA instead of probe molecules is performed. As in MLPA, a universal set of primers is used for the amplification therefore providing similar amplification efficiencies. In this work, the potential of MLPA was probed for the simultaneous amplification of three GMOs maize events (MON810, GA21 and MON863) and the maize reference *adh* gene. The resulting amplicons were analyzed by CGE using 2-hydroxyethyl cellulose acting as sieving matrix and dynamic capillary coating. The high speed and sensitivity of the CGE-LIF was very useful for the optimization of the MLGA conditions. The method was successfully applied for the simultaneous detection of GMOs in maize samples with LODs of 0.2% for GA21, 0.3% for MON863 and 0.1% for MON810. In a further work, the same group described an enzymatic procedure for the production of long DNA probes for their use in MLGA-CGE-LIF for the detection of GMOs in maize [26]. Oligonucleotides are generally obtained by chemical synthesis but this approach is inefficient producing long oligonucleotides leading in general to mixtures of shorter oligonucleotides [27]. As an alternative, in this work, an enzymatic procedure based on ligation of short DNA sequences using a short oligonucleotide that acts as bridge stabilizing the molecular complex was employed for the synthesis of long DNA probes. These probes were employed for the MLGA-CGE-LIF analysis of GM maize (MON863). Using the same CGE-LIF method and similar MLGA conditions as in the previous work [25], the MLGA-CGE-LIF method was employed for the analysis of MON863 using the probe obtained by DNA ligation and a synthetic selector probe. Interestingly, 10-fold higher intensities were observed in the case of the in-lab prepared probe than for the automatic chemical synthesis indicating much stronger amplification. Contents lower than 1% were sensitively detected with this method but a real LOD for the detection of MON863 in maize samples was not established. These results indicated the potential of combining MLGA-CGE-LIF using ligation-based approaches for selector probe production for the determination of GMOs.

In addition to maize, PCR products of genetically modified cultivars as soybean [28], cotton [28,29] and yeast [30] have been analyzed using CGE with LIF detection. Genetically modified yeasts (EKD-13 *Saccaromyces cerivisiae*) have been determined in wines by multiplex PCR and CGE-LIF [30]. Recombinant EKD-13 *Saccaromyces cerivisiae* has been designed to increase the release of mannoproteins to media during the fermentation [31] and was selected as the model strain. The extraction of the yeast DNA from the wine samples was a critical step since yeast DNA was not clearly detected on initial experiments. Therefore, the extraction method was carefully optimized and the use of a commercial kit (MasterPure, Epicentre Biotechnologies) with some modifications, including the addition of polyvinylpolypyrrolidone (PVPP), was selected to properly extract the yeast DNA from the wine samples. Using multiplex PCR, two specific segments of the transgenic construction were amplified and analyzed by CGE-LIF in order to unequivocally detect the transgenic yeast. Two new primers were designed for this purpose and a third primer pair was included as control for the amplification of the *mrp2* gen for *S. cerevisiae* genome. Good reproducibility was obtained for the CGE method with relative standard deviation (RSD) values lower than 2% and 4% for migration times and DNA size determination, respectively. The PCR-CGE-LIF method was successfully tested by analyzing wine samples obtained in different vinification processes. A multiplex PCR-CGE-LIF assay was developed for the detection of cotton and five GM events (MON531, MON15985, MON1445, 3006-210-23 and 281-24-236) [29]. Primers labeled with tetrachloro-6-carboxy-fluorescein (TET), hexachloro-fluorescein (HEX), or FAM™ were employed to obtain good sensitivity. The assay was able to detect up to 0.1% of GMO even in presence of high amounts of other targets. The method showed good specificity allowing to correctly classifying ≥98.3% of samples per target. The assay was combined with a second PCR assay to allow the simultaneous detection of the six cotton targets with four GM maize events (Mon810, Bt11, GA21 and NK603) and a maize endogenous reference gene (*adh1*). As a consequence of the high resolution power obtained in the CGE method, it was possible to analyze the PCR products of the two multiplex PCR using a single CGE method.

Three different screening PCR-CGE methods were developed for the detection and identification of *Bacillus thuringiensis* (Bt) cotton and roundup ready soybeans (RRS) [28]. Four new primers containing fluorescent dyes (6-FAM for *Nos* terminator (t*Nos*), Sad1 and Cry1Ac and HEX for *35S* promoter (p*35S*)) were designed in this work. First, the effectiveness of a duplex methodology for the determination of p*35S* and t*Nos* terminator in cotton was demonstrated. Really good sensitivity was obtained with this method allowing to detect up to 0.01% of GMO in cotton. In a second methodology, a multiplex PCR-CGE method was developed to determine t*Nos* and p*35S* together with *Sad1* and *Cry1Ac* in cotton. Also, good sensitivity was obtained with this method allowing to detect up to 0.01% of *Cry1Ac* and 0.05% of t*Nos*, p*35S* and *Sad1*. These LODs are comparable with that obtained with real time PCR methods. To test the performance of these new primers, an interlaboratory validation was carried out. From the 120 samples analyzed, only three false-positive and two false-negatives were reported. Finally, p*35S* and t*Nos* and the endogenous reference *Lec* were determined in soybean using singleplex PCR-CGE. The sensitivity in this case was 0.1% of GMO in the soybean samples.

**Figure 1:**
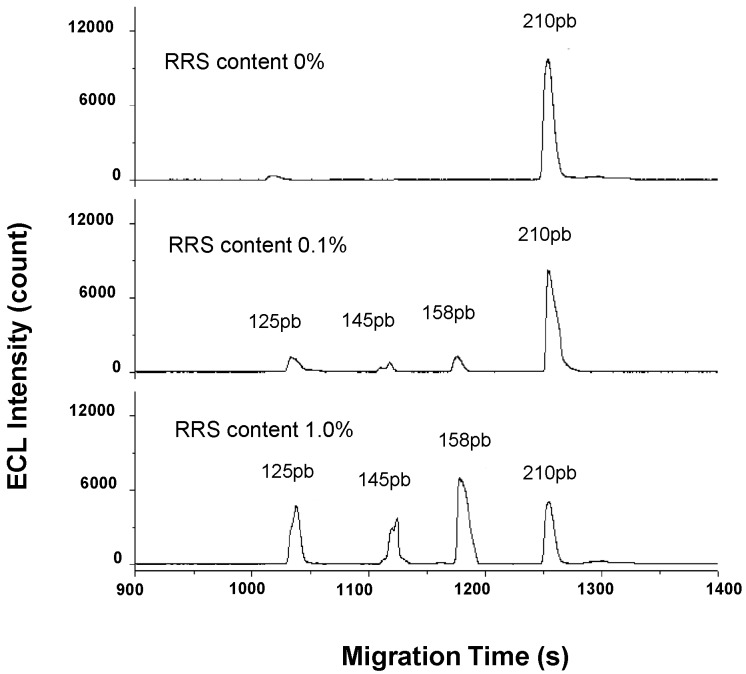
Electrophoregrams obtained by CE-electrochemoluminiscence (ECL) after multiplex PCR for roundup ready soybeans (RRS) certified reference material standards containing 0%, 0.1% and 1.0% of RRS. Reprinted with permission from [33]

As can be seen in Table 1, besides fluorescence detection, CGE has been employed in combination with other detection systems for the detection of PCR products [32,33,34]. Jiang *et al.* reported a methodology for the determination of PCR amplified DNA fragments from transgenic soybeans using CE in its simplest mode (capillary zone electrophoresis, CZE) with UV detection [32]. In order to extract DNA from the soybeans in a fast, simple, less harmful and effective way, a new solid phase extraction method with magnetic particles (MSPE) was developed. Chitosan functionalized magnetic particles were employed for this purpose. DNA can be captured and released easily from the chitosan particles by changing the pH and therefore aqueous solutions can be employed. Good purity of the DNA extracted was obtained after optimization of the extraction procedure. The extracts were directly employed for PCR amplification of t*Nos* and p*35S* events. The PCR products were analyzed by CZE-UV using uncoated capillaries and a background electrolyte (BGE) consisting of 2 mM EDTA and 20 mM Tris–phosphoric at pH 7.3 simplifying and facilitating the detection of GMs in soybeans. In a really interesting work, Guo and collaborators proposed for the first time the use of CE with electrochemoluminiscence (ECL) for the detection of PCR products [33]. ECL detection is a special CL mode where chemical luminescence emission is generated in the process of substance oxidation and reduction at an electrode surface. In this work, the ECL luminophore was employed to label the PCR amplicons allowing the detection of DNA molecules which have no co-reactant functionalities and cannot be detected by conventional ECL. RR soybean was employed as model for the study of the new approach and four target sequences were amplified during the multiplex PCR (35S, t*Nos*, *cp4-epsps* and *lectin*). Tris(1,10-phenanthroline ruthenium(II) (Ru(phen)_3_^2+^) was selected as lumiphore and the label of the primers was investigated. No side effect was observed during amplification. The labeled primers showed good stability and efficiency during amplification. After optimization of the PVP content, that acts as inner dynamic coating as well as sieving matrix for DNA segments separation, good resolution was obtained for the four amplicons. Figure 1, shows the electropherogram obtained under optimal conditions for the detection of soy samples containing different amounts of GM. As it can be observed, good resolution and excellent sensitivity (0.01% GMO) was obtained with this novel method. In another work, it was also demonstrated for the first time, the potential of the combination of CGE with capacitively coupled contactless conductivity detection (C^4^D) for the analysis of PCR products [34]. C^4^D presents advantages as the non-necessity of light absorption by the analytes, easy handling, reduced background noise (in comparison with normal conductivity detection) and robustness. Therefore, labeling of the primers to provide absorbing amplicons is not necessary with this detection system. Bacterial plasmid DNA and transgenic soybeans (RRS) were selected to demonstrate the applicability of the CGE-C^4^D system for the detection of DNA products. PVP was also used in this work as sieving matrix for DNA separation because of its electrical neutrality (necessary for C^4^D detection) and relative low viscosity. Good separation was obtained using a BGE consisting of 20 mM Tris/Ches at pH 8.5 for 5 DNA fragments between 100 and 2000 pb and eight PCR products from bacterial plasmid. A 400 pb length PCR product from RRS was also analyzed showing the applicability of the proposed methodology for GM analysis.

**Figure 2:**
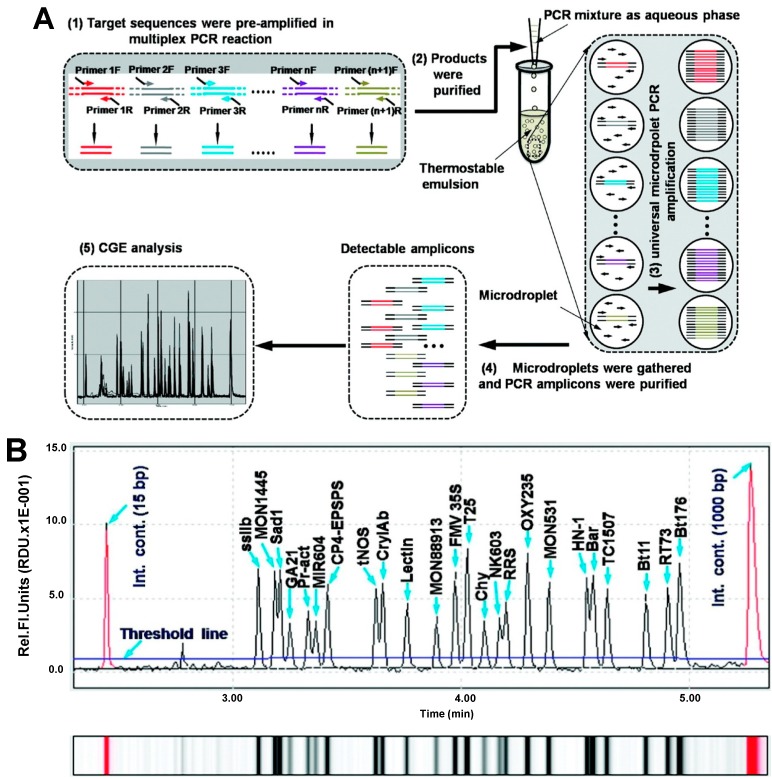
(**A**) Schematic diagram of the multiplex Microdroplet PCR Implemented Capillary gel electrophoresis (CGE) (MPIC) assay. (1) Target-specific DNA products with universal tails on each side were generated in multiplex PCR preamplification, using bipartite primers; (2) The products of the multiplex preamplification were purified and used as templates in the second step, universal microdroplet PCR amplification, using universal primer pairs; (3) The microdroplet PCR was performed, employing the universal primer pair Uni-F/R in the emulsion droplets; (4) The w/o emulsion was centrifuged to gather all microdroplets, and DNA fragments were purified for subsequent CGE-based detection; (5) Detectable amplicons were analyzed by CGE; (**B**) Capillary gel electrophoresis analysis of the 24-plex MPIC assay for detection of 14 different GM events using 24 bipartite primer pairs. Amplicons were produced from a GM DNA solution mixture containing 1.6% of each of the 14 different GM events. The peaks of the amplicon are indicated by blue arrows. The profile shown under the electrophoregram is the simulated gel image corresponding to the results of the capillary gel electrophoresis analysis. Reprinted with permission from [36].

The detection and identification of GMOs in unknown samples was reported by Ryamond *et al.* using a DNA insert fingerprinting in combination with a sensitive CGE-LIF method [35]. The DNA fingerprinting methodology was based on the use of a restriction enzyme digestion, an adaptor ligation and a nested PCR followed by the corresponding CGE-LIF analysis. Fingerprinting the transgenic sequence elements toward the inserted gene, the DNA insert fingerprint provides a characteristic pattern for the GMO construct that may be used to identify known and unknown inserted genes. Therefore, as restriction enzymes will cut at different positions in the gene insert, the fingerprint pattern will vary due to differences in sequences. The PCR products were analyzed using a four-capillary array. The fingerprinting was performed for 12 different maize events and one soy event and the fingerprinting profiles were included in a database that can be further employed for the identification and characterization of the GMOs in unknown maize samples. The method showed good inter-laboratory reproducibility with differences in fragment size below 2.4 bp. The main drawback of the method was the low sensitivity obtained (~1% GMO) but the authors reported that further optimization of the multiplex primer set and conditions of the DNA insert fingerprint method may increase the sensitivity (~0.1% GMO). Table 1 shows that multiplex approaches in combination with CE are really useful for the simultaneous amplification and identification of multiple GMO targets. However, as the possibility of artifacts as a consequence of cross-amplification and/or non-specific amplification increases with the number of added primer pairs, the number of multiple PCR amplification is generally limited to a maximum of 10 targets. In a really interesting work, Guo and collaborators developed a high-throughput method for multiple DNA targets based on multiplex Microdroplet PCR Implemented CGE (MPIC) [36]. For this purpose, as it can be observed in Figure 2a, each target DNA is preamplified using a short number of cycles (11 cycles) by multiplex PCR with bipartite primers containing the same set of universal sequences tails. Then, the preamplified DNA products are purified and used as templates for the microdroplet PCR amplification which can be amplified in parallel using a universal common primer. In addition, as the number of templates DNA molecules is smaller than the number of emulsified microdroplets, no more than a single DNA template will be present in each microdroplet. Therefore, multiple DNA target molecules can be simultaneously amplified with high efficiency and specificity using this approach. In combination with the high efficiency and resolution offered by CGE compared with conventional gel electrophoresis, this approach allows to identify a high number of target sequences in a fast and simple way. To test the proposed system, 24 different target DNA fragments from 14 different GM events were analyzed. As it can be observed in Figure 2b, adequate resolution was obtained for the DNA target, some of them differing in only 5 pb. LODs of 0.1% of GMO were obtained for all the targets with the proposed methodology.

**Table 1:** Applications of capillary electrophoresis (CE) to the determination of DNA in transgenic cultivars.

Cultivar	Target DNA	DNA Amplification Approach	CE Mode	Detection (LOD)	CE Conditions	Ref.
Maize	DAS59122, LY038, MON88017, MIR604, event 3272 and *hmga* gene	Multiplex PCR	CGE	LIF (0.1%)	capillary, 50 cm × 50 µm containing POP-7™ as gel; voltage, 15 kV; temperature, 60 °C; injection, 15 s	[21]
Maize	DAS59122, LY038, MON88017, MIR604, Event 3272 and *hmga* gene	Multiplex PCR	CGE	LIF (0.1%)	capillary, 50 cm × 50 µm containing POP-7™ as gel; voltage, 15 kV; temperature, 60 °C; injection, 15 s	[22]
Maize	TC1507, MON810, NK603, MON863, BT176, T25, GA21, BT11 and *hmga* gene	Ligation-mediated probe amplification	CGE	LIF (0.4%–0.5%)	capillary, 50 cm × 50 µm containing POP-7™ as gel; voltage, 15 kV; temperature, 60 °C; injection, 15 s	[23]
Maize	MON810, GA21, MON863 and *adh* gene	Ligation-mediated genome amplification	CGE	LIF (0.1%–0.3%)	capillary, 50cm × 75 µm; BGE, 20 mM Tris, 10 mM phosphoric acid, 2 mM EDTA and 4.5% HC at pH 7.3; voltage, −13 kV; temperature, 45 °C; injection, 0.5 psi × 40 s	[25]
Maize	MON863	Ligation-mediated genome amplification	CGE	LIF (n.i)	capillary, 50cm × 75 µm; BGE, 20 mM Tris, 10 mM phosphoric acid, 2 mM EDTA and 4.5% HC at pH 7.3; voltage, −13 kV; temperature, 45 °C; injection, 0.5 psi × 40 s	[26]
Yeast	*KmP*, *KmT* and *mrp2*	Multiplex PCR	CGE	LIF (n.i.)	capillary, 50 cm ×75 µm; BGE, 20 mM Tris, 10 mM phosphoric acid, 2 mM EDTA, 500 nM YOPRO-1 and 4.5% HEC at pH 7.3;voltage, −13 kV; temperature, 45 °C; injection, 0.5 psi × 40 s	[30]
Cotton	MON531, MON15985, MON1445, 3006-210-23 and 281-24-236 and *adh1* gene	Multiplex PCR	CGE	LIF (0.1%)	capillary, 47 cm × 50 µm containing POP-4™ as gel; voltage, 15 kV; temperature, 60 °C; injection, 5 s × 15 kV	[29]
Cotton	p*35S*, t*Nos*, *Sad1* and *Cry1Ac*	Multiplex PCR	CGE	LIF (0.01%–0.05%)	capillary, 36 cm × 50 µm containing POP-4™ as gel; voltage, 15 kV; temperature, 60 °C; injection, n.i.	[28]
Soybean	p*35S*, t*Nos*, and *Lec*	Singleplex PCR	CGE	LIF (0.1%)	capillary, 36 cm × 50 µm containing POP-4™ as gel; voltage, 15 kV; temperature, 60 °C; injection, n.i.	[28]
Soybean	t*Nos*, p*35S*	Multiplex PCR	CZE	UV (n.i.)	capillary, 40 cm × 75 µm; BGE, 2 mM EDTA, 20 mM phosphoric acid adjusted at pH 7.3 with Tris; voltage, −8 kV; temperature, 25 °C; injection, 10 kV × 10 s	[32]
Soybean	tt*Nos*, p*35S*, *cp4-epsps*, *lectin*	Multiplex PCR	CGE	ECL (0.01%)	capillary, 45 cm × 75 µm; BGE, 20 mM Tris–HCl, 2 mM EDTA, 1.5 M urea and 2.5% (*w*:*w*) PVP at pH 8.0; voltage, −13.5 kV; temperature, n.i; injection, −13.5 kV × 12 s	[33]
Soybean	400 pb target DNA	Singleplex PCR	CGE	C^4^D (n.i.)	capillary, 45 cm × 75 µm; BGE, 20 mM Tris–HCl, 2 mM EDTA, 1.5 M urea and 2.5% (*w*:*w*) PVP at pH 8.0; voltage, −13.5 kV; temperature, n.i; injection, −13.5 kV × 12 s	[34]
Maize and soybean	Maize, Bt176 (N09K9, MAX40), Bt11 (N44P4, 27M3), G4030, 09A4, 26L6, N4424, T25, MON88017, MON863xNK603 and MON863xNK603xMON810; soybean, GTS 40-30-2	DNA insert fingerprint	CGE	LIF (1%)	Four-capillary array, 36 cm × 75 µM; voltage, 15 kV; temperature 60 °C; injection, 3 kV × 5 s	[35]
Soybean, maize, canola and cotton	Bt176, Bt11, TC1507, NK603, T25, MIR604, GA21, MON531, MON1445, MON88913, RT73, OXY235, RRS, HN-1, *ssIIb*, *Lectin*, *Sad1*, *Chy*, t*Nos*, FMV35S, CP4-EPSPS, *CryIAb*, Bar, Pr-act	Multiplex microdroplet PCR	CGE	Fluorescence (0.1%)	capillary, n.i; BGE, QIAxcel DNA high resolution kit buffer; voltage, 6 kV; temperature, r.t.; injection, 0.5 psi × 20 s	[36]

n.i.: not indicated; r.t.: room temperature.

### 2.2. Protein Analysis

Study and characterization of the proteins present in the GMOs has been fundamental for a better understanding of the effect caused by the genetic modification. Specially, protein profiling of non-targeted proteins has shown to be a good approach to study unexpected modifications in transgenic cultivars [18]. CE offers attractive features for the analysis of proteins due to its high efficiency and the absence of stationary phases that can lead in changes in protein conformation. Another important aspect is that separation is performed under (near-)physiological conditions avoiding protein degradation during analysis [37]. Table 2 shows the recent applications of CE for the determination of proteins in transgenic cultivars. One of the main difficulties of protein analysis in CE is the adsorption of the proteins to the capillary wall. A common approach in CE to prevent protein adsorption is to coat the inner capillary wall with surface coating agents. Pobozy *et al.* evaluate the performance of various capillary coatings for the profiling of fractionated protein extracts from maize samples [38]. Three coatings were tested, including dynamic (hydroxypropylmethylcellulose) and static (polyacrylamide and ω-iodoalkylammonium) coatings. The neutral-coated capillary (polyacrylamide coated capillary) offered the best results in terms of separation efficiency and repeatability of the protein fractions. Because albumins and globulins concentration is low compared with other protein fractions, preconcentration using filters with a cutoff of 3 kDa was necessary to detect the proteins. Comparison of the CE protein profiles obtained for the extracted zein fraction from maize standard samples containing 0% and 5% of Bt11 GMO showed not differences suggesting equivalence of the zein fraction in this specific genetic modification. In the case of the albumins, small quantitative and qualitative differences were found between both samples indicating differences in the content of the albumin fraction of the Bt11 maize in comparison with the isogenic counterpart. In another work, Latoszek *et al.* proposed the use of dendrimers, symmetrical macromolecules with three-dimensional structures, as pseudostationary phases in CE to decrease protein adsorption and improve resolution on the protein profiles increasing the chance of discrimination between conventional and transgenic maize [39]. Silicon-based and dendrimers with amine core were tested in different concentrations. Similar results were obtained with both classes of dendrimers. Protein profiles obtained for ACN/water extracts of a transgenic maize sample and its isogenic counterpart showed clear differences suggesting that the method can be employed for the identification of maize GMOs.

Sázelová *et al.* developed a methodology for the extraction of water-soluble proteins from non-transgenic and transgenic maize Bt and their separation by CZE [40]. The extraction method comprised a shaking and a centrifugation step after addition of water to the grounded maize. The positively charged proteins were separated at strongly acidic BGE in order to reduce the dissociation of silanol groups and therefore suppress the adsorption of the proteins and peptides to the capillary wall. After evaluation of different BGEs the best resolution was obtained with an isolectric BGE consisting of 0.2 M iminodiacetic acid at pH 2.6 allowing to separate 22 peaks. In addition, significant differences between transgenic and not transgenic samples were obtained with this BGE indicating non substantial equivalence of these two cultivars. It is important to mention that the use of a classical acid salt BGE (100 mM H_3_PO_3_, 50 mM Tris pH 2.25), although offering lower resolution (only 12 separated peaks), also allowed to differentiate between conventional and transgenic aristis-Bt.

**Table 2:** Applications of CE in the determination of proteins in transgenic cultivars.

Cultivar	GMO	Analyte	CE Mode	Detection	CE Conditions	Sample Treatment	Ref.
Maize	Bt resistant (Bt11)	Zein fraction	CZE	UV	BGE: 100 mM phosphate buffer pH 3 containing 60% *v*:*v* ethanol; polyacrylamide coated capillary, 40 cm × 50 µm; voltage, 20 kV; temperature, 25 °C; injection, 0.5 psi × 10 s	Extraction with 70% *v*:*v* ethanol with 2% *v*:*v* β-mercaptoethanol	[38]
Maize	Bt resistant (Bt11)	Albumin and globulin fraction	CZE	UV	BGE: 100 mM phosphate buffer pH 3 containing 60% *v*:*v* ethanol; capillary, 40 cm × 50 µm; voltage, 20 kV; temperature, 25 °C; injection, 0.5 psi × 10 s	Albumin fraction: extraction with water and ultrafiltration (3 KDa cut off). Globulin fraction: extraction with 50 mM Tris buffer (pH 7.8) containing 50 mM KCl and 5 mM EDTA and ultrafiltration (3 KDa cut off)	[38]
Maize	Bt resistant (Bt11)	Protein water/ACN (75/25, *v*/*v*) fractions	EKC	UV	BGE: 80 mM phosphate buffer pH 2.5 containing 5% ACN and 0.01% DAB dendrimer; capillary, 40 cm × 50 µm; voltage, 10 kV; temperature, 25 °C; injection, 3.4 KPa × 5 s	Extraction with water/ACN (75:25, *v*:*v*) +s0.3% acetic acid	[39]
Maize	Bt resistant	Water soluble protein fraction	CZE	UV	BGE: 100 mM H_3_PO_4_, 50 mM Tris pH 2.25 or 200 mM iminodiacetic acid pH 2.26: capillary, 20 cm × 50 µm; voltage, 10 kV; temperature, r.t.; injection, 1.2 KPa × 22 s	Extraction with water	[40]
Maize	Bt resistant	Trypsin digested water soluble protein fraction	CZE	UV	BGE: 200 mM iminodiacetic acid pH 2.26; capillary, 20 cm × 50 µm; voltage, 10 kV; temperature, r.t.; injection, 12 mbar × 12 s	Extraction with water and trypsin digestion	[41]
Soybean	Glyphosate resistant (SB10)	Trypsin digested water/ACN (80/20, *v*/*v*) protein fractions	CZE	ESI-MS (+)	BGE: 0.5 M formic acid; capillary, 90 cm × 50 µm; voltage, 25 kV; temperature, 25 °C; injection, 0.5 psi × 20 s	Extraction with water/ACN (80:20, *v*:*v*) and trypsin digestion	[42]

r.t.: room temperature.

**Figure 3:**
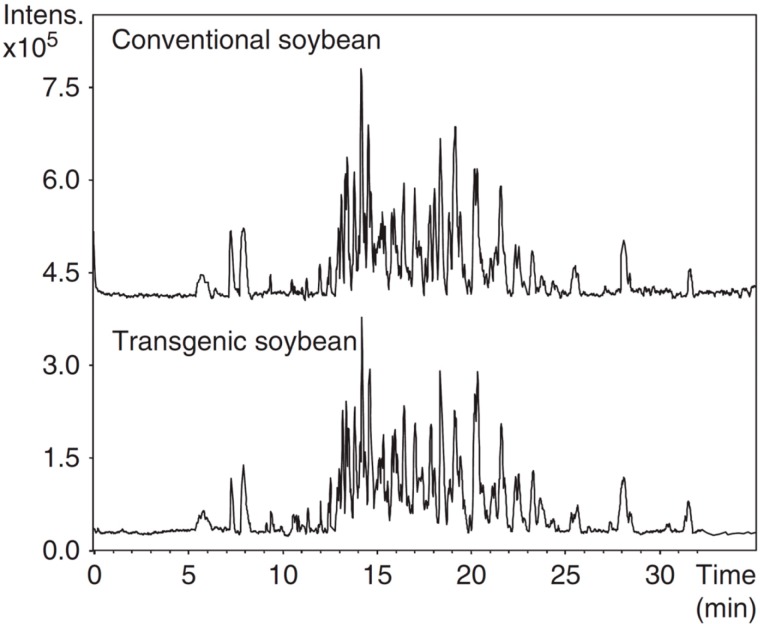
CE-TOF base peak electropherogram of the digested protein extract from conventional and transgenic soybean. Reprinted with permission from [42].

Separation of proteins is not always an easy task especially when isoforms or minority proteins should be determined. Enzymatic hydrolysis of proteins is an alternative to decrease complexity of analysis. Although digestion results in a more complex mixture, peptides are better separated by CE (and other separation techniques) and easily detected by MS where identification of large proteins is challenging. Profiling of peptides obtained after enzymatic digestion of protein cultivars was carried out to study and characterize genetically modified soybean and maize cultivars [41,42]. Bt-transgenic and conventional maize samples were digested using trypsin and the resulted peptides were analyzed by CE–UV [41]. The digestion of the water soluble proteins extracted from the maize was performed using immobilized trypsin in agarose gel. Separation was achieved using two different BGEs, a classical acid/salt-based BGE (100 mM H_3_PO_4_, 50 mM Tris pH 2.25) or an isoelectric BGE (iminodiacetic acid pH 2.26). After comparison of the peptide profiles obtained for a transgenic-BT and an isogenic non-transgenic counterpart, several qualitative and quantitative differences were observed. These results suggest that these peptides could be potentially used for differentiation between these maize species. However, validation with a large set of samples grounded under different conditions is still required. Regarding soybean cultivars, a CE–TOF-MS proteomic approach was developed and employed for the analysis of peptides obtained after trypsin digestion of conventional and genetically modified soybeans [42]. Protein extraction and enzymatic digestion were optimized in order to obtain the higher number of peptides derived from the soybeans. Good peptide profile was obtained by extracting proteins with water:ACN (80:20, *v*:*v*) and digesting under reductive alkylation conditions with trypsin. Peptide separation was reached in less than 30 min using 0.5 M formic acid as BGE. The method was employed for the study of possible unintended effects caused by a genetic modification. In order to carry out an accurate evaluation, only peptides observed in five consecutive injections were considered for the comparison between transgenic and conventional soybeans. A reproducible set of 151 peptides were observed in the five injections. No qualitative changes were observed between the analyzed samples for the 151 peptides evaluated (Figure 3). Although these results suggested the equivalence between GM and non-GM soybeans, more experiments are required to confirm it as some differences were observed for less abundant peptides that were not included in the mentioned study.

### 2.3. Metabolite Analysis

Metabolite determination has proved to be a useful and necessary tool in the study and characterization of GMOs that provides information not only about the differences between non-modified and modified organisms metabolites but also about their different response to diverse situations and/or stress conditions (e.g., environmental, climatic, infections). CE, generally coupled with MS, has demonstrated a good profiling potential towards highly polar and charged metabolites providing complementary information with respect to other applied methodologies as LC-MS [17]. Table 3 summarizes the recent applications of CE for the metabolic study of different GMOs. As it can be expected, excluding one work aimed at the stereospecific determination of amino acids contained in yeast, most applications comprise the use of MS detection, providing the additional information needed for metabolite identification.

**Figure 4:**
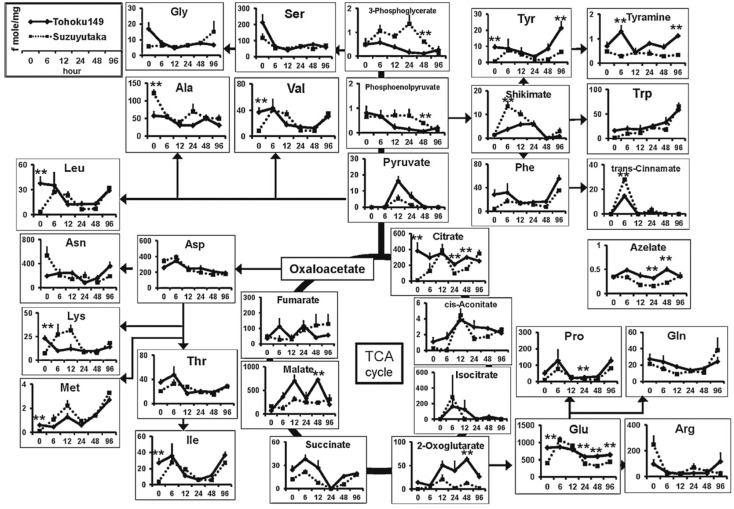
Changes in the level of representative primary metabolites after aphid introduction. Data for the metabolite per mg of soybean leaves (wet weight) at 0–96 h after aphid introduction are plotted. Solid and dashed lines indicate the metabolite concentrations in Tohoku 149 (*n* = 5) and Suzuyutaka (*n* = 5), respectively. The data points are mean values and the error bars indicate the standard error. Asterisk (**) denotes statistical significance with *p* < 0.01. Reprinted with permission from [44].

CE-TOF-MS in combination with FT-ICR-MS has been employed to study the metabolites present in three varieties of Bt-maize and their isogenic counterparts [43]. Although FT-ICR-MS detected a higher number of metabolites, the use of CE-TOF-MS gave additional information based on the electrophoretic mobility for compounds not found in databases. Therefore, the determination of electrophoretic mobility permitted to confirm the tentative assignment made by FT-ICR-MS for 10 of the proposed biomarkers found in transgenic samples and not in the isogenic counterparts or *vice versa*. In addition, metabolite extraction using a pressurized liquid extraction method and different solvents were evaluated providing interesting results that may be combined with the MS-based methods to gain information about the nature of the metabolites present in the samples. An interesting study of the metabolomic response of wild type (Suzuyutaka) and aphid-resistant (Tohoku149) soybeans to aphid exposure by CE-TOF-MS was performed by Sato and collaborators [44]. Plants were submitted to aphids infection and leafs were removed at different times after the introduction of the aphids. The metabolites contained in leafs were extracted with MeOH and analyzed by CE-TOF-MS using uncoated capillaries and an acidic BGE (1M formic acid) for cationic metabolites and cationic coated capillaries (COSMO (+)) and a basic BGE (50 mM ammonium formate pH 8.5) for anionic compounds. Results showed that many metabolites were in higher concentrations in plants with higher resistance to aphids. Interestingly, after aphid introduction these differences were reduced at 6–24 h (See Figure 4). In addition, as it can be observed in Figure 4, the metabolites related to energy metabolism (Krebs cycle) were increased after 48 h in the Tohoku149 suggesting that nicotinamide adenine dinucleotide (NADH) and adenosine triphosphate (ATP) are consumed to recover from damage caused by the aphids. These results proved that CE-MS can be a useful tool for the study of the mechanisms of resistance of cultivars towards aphid infection and to evaluate changes on metabolic responses between transgenic and non-transgenic cultivars. Using a similar approach, the metabolomic response of different rice cultivars towards death-inducible stress was carried out [6]. Conventional rice cultivars and cultivars over-expressing Arabidopsis bax inhibitor-1 (BI-1), a cell suppression factor, were subjected to menadione-induced cell death. Cell death was suppressed by 40%–50% in BI-1-cells. CE-MS was further employed to study the metabolic changes in the oxidatively stressed rice cells. Three different methods comprising acidic, basic and medium pH BGEs were employed for the determination of cationic and anionic metabolites and nucleotides, respectively (See Table 3). The metabolite analysis of the rice cells did not show qualitatively or quantitatively differences under non-stress conditions. After menadione treatment, metabolites involved in pathways as amino acid metabolism, redox states and energy change were altered. Interestingly, metabolic alteration was only observed for the transgenic lines suggesting that cell death suppression is correlated with the capacity of the cell to metabolic acclimation under oxidative conditions. These results demonstrate again the importance of the study of metabolism changes after a genetic modification as the responses to certain situations or stress conditions can vary in comparison with the non-modified counterparts. The same group reported other metabolic study on rice mutants [7]. In this occasion, transgenic rice plants expressing the *Arabidopsis* chloroplastic NAD Kinase (NADK) gene (NK2 lines) were the object of study. This modification stimulates carbon and nitrogen assimilation of the rice plants. Metabolites from the leaves were extracted using an hydroorganic mixture (MeOH:water, 50:50, *v*:*v*) and analyzed by CE-MS. As in the previous work, three different methods were employed for the analysis of the extracted metabolites (See Table 3). The results revealed increased levels of amino acids and sugar phosphates in the NK2 lines. In addition, an enhanced activity of NADK and accumulation of NADP was observed. The tolerance to oxidative stress in KN2 lines was also evaluated. Higher tolerance was observed for the modified lines, suggesting that these lines stimulate photosynthesis metabolism and tolerance to oxidative damages. In other similar study, Suzuky *et al.* used CE-MS to study the effects on metabolism by genetic modification on rice plants with increased or decreased Rubisco content, an enzyme that plays an essential role in photosynthesis [45]. The metabolites were extracted from the leafs with MeOH followed by a liquid–liquid extraction with water:chloroform and ultrafiltration. Cationic metabolites were separated using an uncoated capillary and strongly acidic BGE whereas the separation of anionic metabolites was achieved employing a cationic coated capillary and a basic BGE. The genetic modification affecting the Rubisco content affected different metabolites including amino acids and metabolites related with the photosynthesis and photorespiration of the plant. The plants with increased Rubisco content did not improve light-saturated photosynthesis and the amount of amino acids was found to be higher in comparison with the conventional cultivars. In addition, glyceraldehydes 3-phophate and sedoheputulose 7-phosphate were increased but ATP and adenosine triphosphate (ADP) levels were not affected. On the other hand, photosynthesis was decreased in plants with decreased Rubisco content and accumulation of ribulose bisphosphate together with an increase of ATP, ADP and the amino acids levels was observed. These results indicated that genetic manipulation of Rubisco contents mainly affect the C and N metabolism.

The stereoselective determination of metabolites has also proved to be a powerful tool for the study of the differences between transgenic and non-transgenic cultivars. Giuffrida *et al.* developed a CE-TOF-MS methodology for the study of the enantiomeric content of different amino acids in wild and glyphosate resistant soybeans [46]. As enantiomers of a chiral compound have identical electrophoretic mobility, the use of chiral selectors to achieve the enantioseparation is necessary. In this regard, cyclodextrins (CDs) are the most employed chiral selectors in CE because their broad chiral recognition ability and the wide variety of commercial available CDs derivatives [47]. In this work, three non-commercial derivatives and two native CDs were tested as chiral selectors for 10 fluorescein isothiocyanate (FITC) amino acids derivatives. From them, 3-monodeoxy-3-monoamino-β-CD was selected as it provided the best discrimination power towards the FITC-amino acids. The methodology was applied to the study of the l- and d-amino acids present in transgenic and non-transgenic soybean. The same amino acids were detected in both varieties. However, some differences were observed regarding the stereochemistry of the amino acids. d-Arg was observed in the transgenic but not in the wild soybean. In addition, the amounts of l-Arg and l-Asp were two folds higher in the transgenic variety. Another work published by the same group can be taken as example of the potential of enantioselective determinations in the study of GMOs [48]. In this case, micellar electrokinetic chromatography with LIF detection was employed for the chiral separation and determination of five FITC-amino acids obtained after the autolysis genetically modified yeasts employed in wine elaboration and the isogenic counterparts. Transgenic autolytic yeast strains can be employed for the acceleration of the aging-like characteristics of the wines and therefore, the study of the products released after the hydrolytic degradation, including d- and l-amino acids, is of high value. To achieve the enantiomeric separation, a native cyclodextrin, namely β-CD, was employed in combination with an anionic surfactant (SDS) at high pH. Under these conditions, baseline separation of the five studied FITC-amino acids was achieved in 25 min. The application of the developed methodology to the analysis of the d- and l- amino acids released form the modified and non-modified yeasts revealed faster autolysis of the mutant which released higher amounts of l-amino acids in shorter times. In addition, levels of d-Arg and d-Glu were higher in the non-modified yeast suggesting a pleiotropic effect involving the protein kinase A pathway in the mutant.

**Table 3:** Applications of CE for the determination of metabolites in transgenic cultivars.

Cultivar/Strain	GMO	Analyte	CE Mode	Detection	CE Conditions	Sample Treatment	Ref.
Maize	Bt resistant (PR33P66Bt, tietar Bt, and Aristis Bt)	Cationic metabolites	CZE	ESI-MS (+)	BGE: 0.5% formic acid pH 1.9; capillary, 80 cm × 50 µm; voltage, 20 kV; temperature, r.t.; injection, 0.5 psi × 15 s	Extraction with MeOH:water (50:50, *v*:*v*)	[43]
Soybean	Aphid resistant (Tohoku149 and Suzuyuka)	Cationic metabolites	CZE	ESI-MS (+)	BGE: 1 M formic acid; capillary, 100 cm × 50 µm; voltage, 30 kV; temperature, 20 °C; injection, 5 kPa × 3 s	Extraction with MeOH followed by dilution in water, protein precipitation with chloroform and ultrafiltration (3 KDa cut off)	[44]
Soybean	Aphid resistant (Tohoku149, Suzuyuka)	Anionic metabolites	CZE	ESI-MS (−)	BGE: 50 mM ammonium acetate solution (pH 8.5); COSMO(+) coated capillary, 110 cm × 50 µm; voltage, −30 kV; temperature, 20 °C; injection, 50.8 kPa × 30 s	Extraction with MeOH followed by dilution in water, protein precipitation with chloroform and ultrafiltration (3 KDa cut off)	[44]
Rice	Transformants over-expresing *Arabidopsis* bax inhibitor-1	Cationic metabolites	CZE	ESI-MS (+)	BGE: 1 M formic acid pH 1.9; uncoated capillary, 70 cm × 50 µm; voltage, 20 kV; temperature, 20 °C; injection, n.i.	Extraction with MeOH, chloroform and water (aprox 42:42:16, *v*:*v*:*v*) followed by MeOH evaporation and ultrafiltration (3 KDa)	[6]
Rice	Transformants over-expresing *Arabidopsis* bax inhibitor-1	Anionic metabolites	CZE	ESI-MS (−)	BGE: 50 mM ammonium acetate pH 9.0; FunCap-CE type S capillary, 80 cm × 50 µm; voltage, −30 kV; temperature, 20 °C; injection, 2 psi × 5 s	Extraction with MeOH, chloroform and water (aprox 42:42:16, *v*:*v*:*v*) followed by MeOH evaporation and ultrafiltration (3 KDa)	[6]
Rice	Transformants over-expresing *Arabidopsis* bax inhibitor-1	Nucleotides	CZE	ESI-MS (−)	BGE: 50 mM ammonium acetate pH 7.5; uncoated capillary, 100 cm × 50 µm; voltage, 30 kV + 50 mbar; temperature, 20 °C; injection, 50 mbar × 30 s	Extraction with MeOH, chloroform and water (aprox 42:42:16, *v*:*v*:*v*) followed by MeOH evaporation and ultrafiltration (3 KDa)	[6]
Rice	Transformants over-expresing *Arabidopsis* NADKinase gene	Cationic metabolites	CZE	ESI-MS (+)	BGE: 1 M formic acid (pH 1.9); uncoated capillary, 100 cm × 50 µm; voltage, 30 kV; temperature, 20 °C; injection, 50 mbar × 3 s	Extraction with MeOH:water (50:50, *v*:*v*) followed by ultrafiltration (5 KDa)	[7]
Rice	Transformants over-expresing *Arabidopsis* NADKinase gene	Anionic metabolites	CZE	ESI-MS (−)	BGE: 50 mM ammonium acetate pH 9.0; uncoated capillary, 80 cm × 50 µm; voltage, 30 kV (+0.10 bar after 30 min); temperature, 20 °C; injection, 2 psi × 5 s	Extraction with MeOH:water (50:50, *v*:*v*) followed by ultrafiltration (5 KDa)	[7]
Rice	Transformants over-expresing *Arabidopsis* NADKinase gene	Nucleotides	CZE	ESI-MS (−)	BGE: 50 mM ammonium acetate pH 7.5; uncoated capillary, 100 cm × 50 µm; voltage, 30 kV + 50 mbar; temperature, 20 °C; injection, 50 mbar × 30 s	Extraction with MeOH:water (50:50, *v*:*v*) followed by ultrafiltration (5 KDa)	[7]
Rice	Transformants with OsRBCS2 cDNA	Cationic metabolies	CZE	ESI-MS (+)	BGE: 1 M formic acid; uncoated capillary, 100 cm × 50 µm; voltage, 30 kV; temperature, 20 °C; injection, 50 mbar × 3 s	Extraction with MeOH, chloroform and water (aprox 42:42:16, *v*:*v*:*v*) followed by ultrafiltration (5 KDa)	[45]
Rice	Transformants with OsRBCS2 cDNA	Anionic metabolites	CZE	ESI-MS (−)	BGE: 50 mM ammonium acetate solution, pH 9.0; SMILE(+) coated capillary, 100 cm × 50 µm; voltage, −30 kV; temperature, 20 °C; injection, 50 mbar × 30 s	Extraction with MeOH, chloroform and water (aprox 42:42:16, *v*:*v*:*v*) followed by ultrafiltration (5 KDa)	[45]
Soybean	Glyphosate resistant	Chiral amino acids	EKC	ESI-MS (+)	BGE: 50 mM ammonium hydrogen carbonate pH 8.0 containing CD_3_NH_2_; capillary, 85 cm × 50 µm; voltage, 30 kV; temperature, 25 °C; injection, 0.5 psi × 25 s	Extraction with 0.37 M trichloroacetic acid and 3.6 mM sodium deoxycholate followed by derivatization with FITC	[46]
Yeast	Autolytic strains (LS11)	Chiral amino acids	EKC	LIF	BGE: 50 mM ammonium hydrogen carbonate pH 8.0 containing CD_3_NH_2_; uncoated capillary, 85 cm × 50 µm; voltage, 30 kV; temperature, 25 °C; injection, 0.5 psi × 25 s	Extraction with 2 M trichloroacetic acid and 3.6 mM sodium deoxycholate followed by derivatization with FITC	[48]

n.i.: not indicated; r.t.: room temperature.

## 3. Applications of Chip-Based Electrophoresis to the Study and Characterization of Transgenic Cultivars

In recent years, the interest in the development of chip-based analytical systems has increased considerably because the inherent merits such as analysis speed, increased performance, small size, and low sample/solvent consumption [49,50,51]. Electrophoresis using microfluidic chips is an alternative to conventional CE since the higher capacity of heat dissipation allows applying higher electric fields yielding higher performance and accelerated separations.

Numerous microfluidic devices have been designed and successfully employed for pharmaceutical, clinical, environmental, and food analysis [52]. A broad number of designs and types of chips are now available in the market. In addition, different companies have developed fully automated microfluidic systems that can be used as a fast and simple alternative for the analysis of proteins and DNA. Some examples are the Caliper LabChipCXII form Perkin Elmer, the BioAnalyzer from Agilent Technologies that can analyze up to 12 samples per run or the MCE-202 MultiNA from Shimadzu, that present higher sample throughput (it can use eight or 12 strip tubes or 96 well plates).

Chip-based electrophoresis has also been employed in the characterization of genetically modified cultivars. In the period covered in this review, few examples have been found in the literature. In an interesting article, Burrell *et al.* performed a comparative analysis of three microchip instruments for the determination of PCR-derived products for the identification of GMOs in soy and maize [53]. Two capillary-based microfluidic instruments (Agilent Bioanalyzer 2100, Agilent Technologies and Shimadzu MCE-202 MultiNA, Shimadzu Corporation) and a gel electrophoresis system (Lab901 TapeStation, Lab901 Limited) were tested for this purpose. An established GMO multiplex approach was employed for the comparison. This approach has extensively been evaluated using the Agilent Bioanalyzer and comprises the simultaneous detection of GM in soy and maize and cauliflower mosaic virus in food samples [54]. The Agilent Bioanalyzer produced the most precise results in the estimation of the amplicon size whereas the Lab901 TapeStation offered the least bias form the theoretical size of the amplicons. In this study, it seems than both, the Agilent Bioanalyzer and the MultiNA overestimated amplicon sizes according to the theoretical size. On the other hand, the better repeatability of the amplicons between runs was obtained with the MultiNA. In general, the three instruments detected correctly the presence or absence of the different amplicons for a range of samples and controls. The Agilent Bioanalyzer has also been employed for the detection of DNA fragments of genetically modified rice [55]. During the research performed by Kluga and collaborators, two real-time PCR methods were compared and evaluated for the detection of different events used in rice modification. First, the use of SYBR^®^ Green (a DNA binding dye that binds only to dsDNA) and real-time PCR for the detection of 35S promoter (P35S), the nopaline synthase terminator (T-nos) and the CryIAb/Ac toxins and second, the use of P35S/T-nos duplex TaqMan^®^ real-time PCR. Both methods detected correctly the target in different control materials. The DNA fragments obtained by these methods were analyzed by PCR, melting analysis and electrophoretic chips. From these results it could be concluded that the use of SYBR^®^ Green resulted in a lower specificity but in a higher GM coverage compared with the duplex TaqMan^®^ method.

Protein profiles obtained by chip-electrophoresis have also been employed to investigate GMOs in maize samples [56]. For this purpose, albumin, globulin and zein fractions were analyzed using two gel chips in parallel with different mass ranges (5–80 and 14–230 kDa). Several proteins were found in significant different amounts in non-transgenic maize samples and samples containing a 5% of GMO. Different sample preparation methods as ultracentrifugation, lyophilization and the use of combinatorial peptide ligand libraries (CPLL) were evaluated to improve sensitivity and selectivity of the method. The CPLL is a sample pretreatment step effective on the determination of low-abundance proteins from complex protein extracts. This method consists of the use of a CPLL sorbent bed in a SPE cartridge that retains in similar amounts proteins that are present at very different concentration levels. Several differences were observed for protein profiles of conventional and Bt-transgenic maize. In particular, triosephosphae isomerase (25.3 KDa), and other two proteins of 6.0 and 20.6 KDa were proposed as potential markers for the identification of GMOs. In addition, the presence of the Cry1Ab protein, the protoxin expressed in Bt maize, was investigated. However, no qualitative differences were observed for this protein in conventional and Bt-transgenic cultivars.

**Figure 5:**
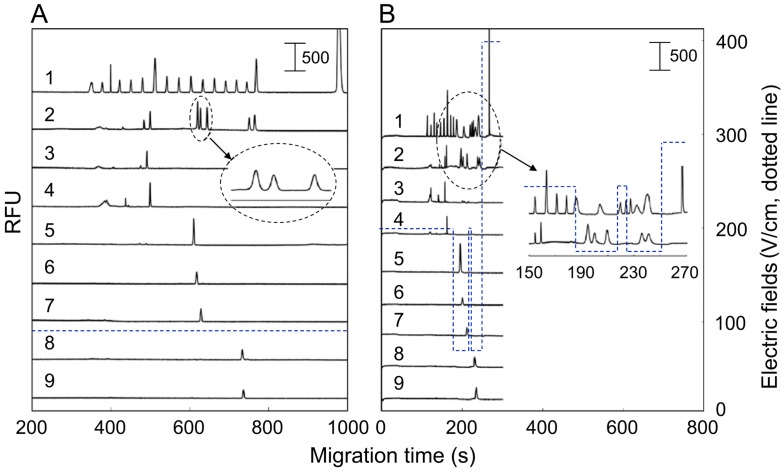
Representative electropherograms obtained by multi-channel microchip electrophoresis of rice knockout DNA sample sunder (**A**) constant field strength (CFS) and (**B**) programmed field strength gradient (PFSG). Reprinted with permission from [57].

In another work, Nan and collaborators employed a lab-made multi-channel microchip electrophoresis with a LIF detector for the detection of knockout mutants encoding pectinase in genetically modified rice [57]. A glass chip containing three parallel separation channels was evaluated for the high-throughput screening of amplified-PCR products. In order to perform the simultaneous detection in the three channels, an expanded laser bean for excitation was employed and the electrodes were designed specifically for this purpose. In total, 0.7% poly(ethylene oxide) (PEO) was selected as sieving gel matrix. A programmed field strength gradient (PFSG) was successfully employed in the three channels to speed up separation without significant loss of resolving power in the region of interest. Figure 5 shows the electropherograms obtained for a 50-pb DNA ladder and different rice mutants obtained by applying constant field strength (CFS) (Figure 5a) and a PFSG (Figure 5b). It can be observed that the PFSG method provides analysis around five times faster than the conventional method whereas maintaining separation of the target DNA fragments. The combination of the PFSG method with a three-channel microchip represents a valuable tool for high throughput screening of rice mutants.

## 4. Conclusions

CE has proven to be a very useful tool for the characterization of transgenic cultivars. The versatility of CE allows the analysis of multiple targets including, DNA, proteins, peptides and metabolites of high importance in the study of GMOs. As a consequence of its high specificity DNA based methodologies are still the preferred methods for the determination of GMOs in crops and food-derived products. The use of gel electrophoresis for the determination of DNA fragments after amplification is being replaced by the use of CGE with LIF detection as a consequence of its high resolution and sensitivity compared with conventional gel electrophoresis. In addition, new designs on instrumentation combining multiple capillary arrays are allowing the high-throughput analysis of these samples. Proteins are another important target in the study and characterization of transgenic cultivars. CE has proved to be a suitable alternative for the analysis of both intact and digested proteins obtained from different modified crops. The investigation of the metabolic response of GMOs to diverse situation or stress conditions (e.g., environmental, climatic, infections) has successfully been performed using CE in combination with MS and has proven to offer complementary information to conventional metabolomic techniques. The capacity of CE to carry out chiral separations has also been exploited in the characterization of transgenic cultivars offering interesting results about the stereochemistry of amino acids in modified samples. Finally, the use of microfluidic chip electrophoresis has shown to be a promising alternative for the fast, effective, and *in situ* analysis of transgenic cultivars.
